# CT measurement of Segond fracture fragment distance to the Gerdy tubercle predicts combined anterolateral ligament and iliotibial band capsulo-osseous layer involvement on MRI

**DOI:** 10.1007/s00256-026-05346-5

**Published:** 2026-08-18

**Authors:** Aonan Wang, Ming Ni, Kun Yang, Qiang Zhao, Ying Liu, Huishu Yuan

**Affiliations:** Department of Radiology, Peking University Third Hospital, 49 North Garden Road, Haidian District, Beijing, 100191 China

**Keywords:** Segond fracture, Gerdy tubercle, Anterolateral ligament, Iliotibial band, Anterior cruciate ligament

## Abstract

**Objective:**

Segond fractures are avulsion fractures of the anterolateral tibial plateau, but the ligamentous structures involved remain debated. This study aimed to determine whether CT-measured fragment distance to the Gerdy tubercle predicts combined involvement of the anterolateral ligament (ALL) and the capsulo-osseous layer of the iliotibial band (ITB) as defined by MRI.

**Materials and methods:**

We retrospectively analyzed 86 patients (58 male, 28 female; mean age 30.9 ± 11.4 years) with acute Segond fractures on CT and MRI. CT measured fragment dimensions and distance to the Gerdy tubercle. MRI assessed the ALL, ITB, and its capsulo-osseous layer, classifying patients into ALL-only (n = 48) vs combined ALL + ITB involvement (n = 38). ROC analysis evaluated CT parameters.

**Results:**

Anterior cruciate ligament(ACL) injury occurred in all 86 patients (100%). MRI showed ALL attachment in 100% and combined involvement in 44.2%. Fragment dimensions did not differ between groups (all *p* > 0.05). CT distance to Gerdy tubercle was shorter in the combined group (9.2 ± 1.6 mm vs 11.2 ± 3.1 mm, *p* = 0.029). A cutoff < 10 mm predicted combined involvement (sensitivity 78.9%, specificity 62.5%; AUC = 0.72, 95% CI 0.60–0.84).

**Conclusion:**

Segond fractures consistently involve the ALL. Combined involvement of the capsulo-osseous layer adjacent to the ITB occurs in a substantial subset and is associated with a fragment distance of < 10 mm from the Gerdy tubercle on preoperative CT, as identified on MRI. This simple, reproducible CT marker may assist in surgical planning for anterolateral complex injuries when interpreted alongside MRI findings.

## Introduction

Segond fracture, first described by French surgeon Paul Segond in 1879 [1], is an avulsion fracture of the anterolateral aspect of the tibial plateau. This injury typically results from a combination of varus stress and internal rotation during knee trauma and is highly associated with concomitant ligamentous injuries, particularly anterior cruciate ligament (ACL) tears [2].

The anterolateral complex (ALC) of the knee comprises the anterolateral ligament (ALL), the iliotibial band (ITB), and the adjacent joint capsule. This complex plays a critical role in maintaining rotational stability of the knee [3]. An international consensus group has further defined the ALC as consisting of the superficial and deep aspects of the ITB with its Kaplan fiber attachments on the distal femur, along with the ALL as a capsular structure within the anterolateral capsule [4]. The ALL—recharacterized in modern anatomical studies by Claes et al. [5, 6] and further defined through systematic dissection techniques [7]—courses from the lateral femoral epicondyle (with its origin being posterior and proximal to the lateral epicondyle) to a tibial insertion just posterior to the Gerdy tubercle [8], closely corresponding to the typical location of Segond fractures. The ITB inserts at the Gerdy tubercle and, via its capsulo-osseous layer, has a broad attachment to the lateral tibial plateau [9]. Prior imaging studies have been limited by the lack of quantitative spatial analysis correlating fragment location with specific ligamentous attachments. A recent MRI-based study by Helito et al. [10] demonstrated that both the ALL and ITB can be attached to Segond fracture fragments, but did not provide a quantitative imaging parameter to predict injury patterns.

Despite extensive research over the past decade [11], the precise capsuloligamentous origin of Segond fractures remains controversial. While several studies have suggested that the fracture represents a bony avulsion of the distal ALL attachment [5, 12, 13], others have proposed involvement of the ITB or the anterolateral capsule [4, 14]. This ambiguity has direct clinical implications: accurate preoperative identification of which structures are injured is essential for determining whether isolated ACL reconstruction suffices or whether additional anterolateral stabilization procedures (e.g., ALL reconstruction, ITB tenodesis, or Segond fragment fixation) are indicated [15].

Prior imaging studies have been limited by the lack of quantitative spatial analysis correlating fragment location with specific ligamentous attachments. The close anatomical relationship among the ALL, the capsulo-osseous layer of the ITB, and the Gerdy tubercle often leads to ambiguous interpretation of apparent "ITB involvement" on MRI.

Therefore, the purpose of this study was to determine whether CT-measured fragment distance to the Gerdy tubercle predicts combined involvement of the ALL and the capsulo-osseous layer of the ITB as defined by MRI, and to identify a reproducible imaging marker to guide surgical decision-making. We hypothesized that Segond fracture fragments located closer to the Gerdy tubercle on CT would be associated with combined involvement of the ALL and the capsulo-osseous layer of the ITB on MRI, and that a specific distance cutoff could be identified to predict this combined injury pattern.

## Materials and methods

### Study population

**Fig. 1:**
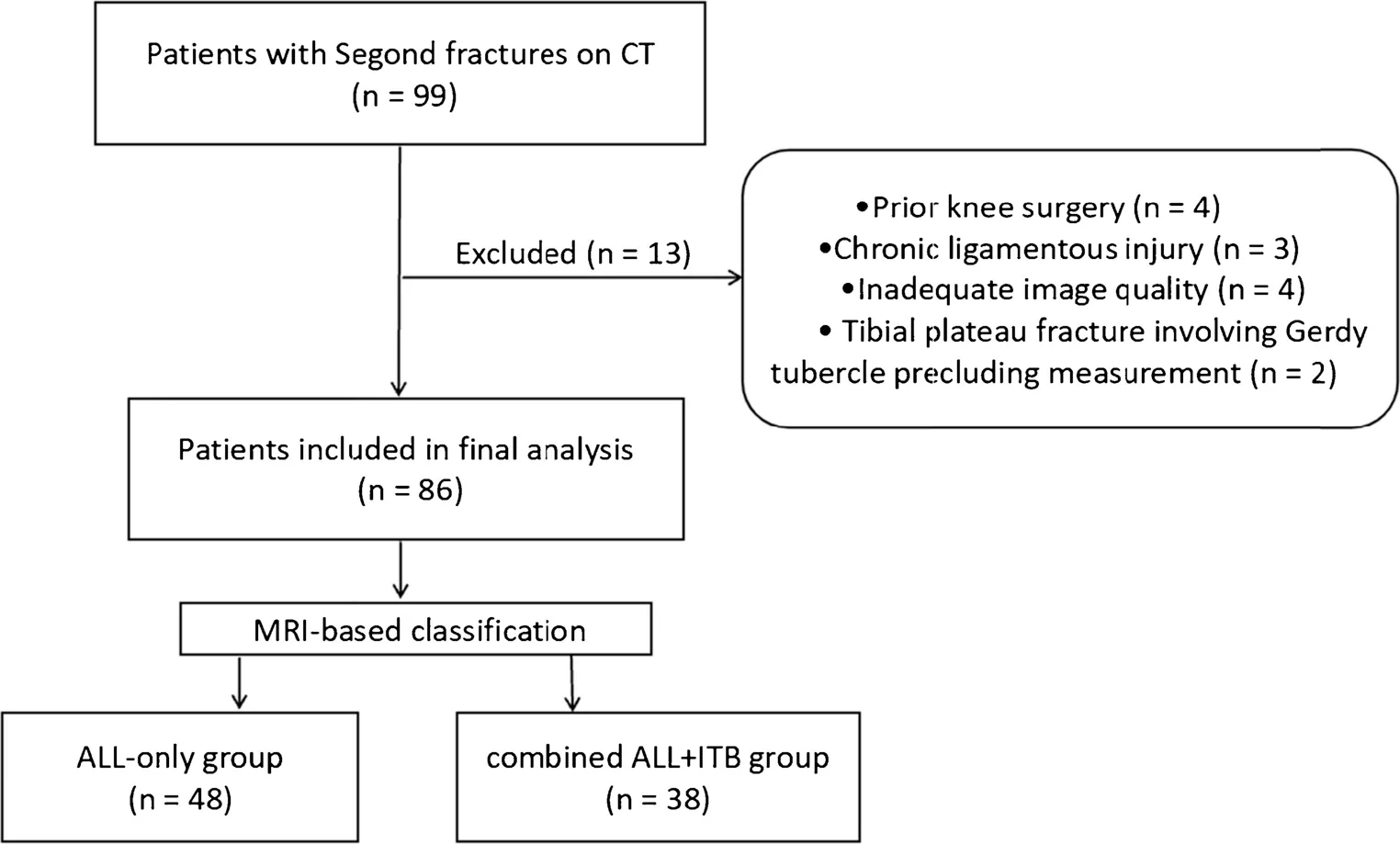
Flow diagram of patient selection and group allocation. (ALL, anterolateral ligament; ITB, iliotibial band)

This retrospective study was approved by the Institutional Review Board of Peking University Third Hospital, and the requirement for informed consent was waived due to the retrospective nature of the study.From August 2023 to January 2026, a consecutive series of patients diagnosed with Segond fractures on CT who also underwent MRI within two weeks of trauma were identified from our institutional database. Inclusion criteria were: (1) acute knee trauma with CT confirmation of a Segond fracture; (2) MRI examination performed within two weeks of trauma; (3) age ≥ 10 years. Exclusion criteria were: (1) history of prior knee surgery; (2) chronic ligamentous injury; (3) inadequate image quality for analysis; (4) associated tibial plateau fracture precluding measurement. During the study period, 99 patients with Segond fractures were initially identified. Of these, 13 were excluded: 4 had a history of prior knee surgery, 3 had chronic ligamentous injury, 4 had inadequate image quality for analysis, and 2 had associated tibial plateau fractures precluding measurement. The remaining 86 patients (58 male, 28 female; mean age 30.9 ± 11.4 years; range 10–68 years) met all criteria and were included in the final analysis. The flow of participants through the study is shown in Fig. 1.

### Imaging protocols

All CT examinations were performed using a spiral CT scanner (uCT 820, United Imaging Healthcare, Shanghai, China) with the following parameters: tube voltage 140 kV; tube current 149 mA; pitch 1.0; slice thickness 1.0 mm. Axial images were reconstructed using a high-resolution bone algorithm and transferred to a dedicated workstation for multiplanar reconstruction (MPR). Standardized bone window settings (width 1500 HU, level 300 HU) were used for all measurements.

All MRI examinations were performed using a 3.0-T MRI scanner (uMR 790, United Imaging Healthcare, Shanghai, China) with a 24-channel dedicated knee coil. The imaging protocol included: fast spin-echo proton density-weighted images (PDWI) in sagittal, axial, and coronal planes (TR/TE 2078–2774 ms/29–32 ms); and T1-weighted images in the sagittal plane (TR/TE 406 ms/9 ms). Slice thickness was 4 mm with a 0.4 mm interslice gap, and field of view (FOV) was 160 × 160 mm. No contrast agent was administered.

### Imaging evaluation

All CT and MRI data were anonymized prior to analysis. Three musculoskeletal radiologists (with 5, 9, and 30 years of experience, respectively) independently reviewed all cases. Each reader performed two separate evaluations with a minimum interval of two weeks to assess intraobserver reliability. Prior to formal assessment, a two-hour calibration session using ten non-study cases was conducted to standardize diagnostic criteria and measurement procedures. Final decisions were reached by consensus when discrepancies occurred.

#### CT assessment

CT was used to assess the osseous characteristics of Segond fracture fragments. The following parameters were measured using MPR in standardized bone window settings:

- Height (superoinferior dimension, mm): measured on coronal images
- Length (anteroposterior dimension, mm): measured on axial images
- Width (mediolateral dimension, mm): measured on axial images
- Estimated volume (mm^3^): calculated as height × length × width (simplified geometric approximation)
- Distance to Gerdy tubercle (mm): measured on axial images from the anterior margin of the fracture fragment to the center of the Gerdy tubercle (defined as the midpoint of its cortical margin on the axial image showing the most prominent tubercle contour)

For each case, fragment dimensions were independently measured by two readers. The final values were calculated as the mean of the two measurements. If the absolute difference exceeded 2 mm, a third senior reader (30 years of experience) adjudicated.

#### MRI assessment

MRI was used to evaluate associated soft-tissue injuries. The following structures were assessed: anterior cruciate ligament (ACL), posterior cruciate ligament (PCL), medial collateral ligament (MCL), lateral collateral ligament (LCL), medial meniscus (MM), lateral meniscus (LM), anterolateral ligament (ALL), and the iliotibial band (ITB) with its adjacent capsulo-osseous layer.

ALL injury was defined as: (1) discontinuity of the ligament along its expected course from the lateral femoral epicondyle to the tibial insertion just posterior to the Gerdy tubercle; (2) abnormal intraligamentous signal intensity on fluid-sensitive sequences; or (3) visible attachment of the avulsed fracture fragment to a soft-tissue structure compatible with the ALL [16]. For ALL injury grading, the classification proposed by Muramatsu et al. [17] was referenced, with category A (continuous), B (warping/thinning/iso-signal), and C (discontinuity) corresponding to normal, edema/contusion, and tear, respectively (Table 1).

**Table 1:** Associated ligament and meniscus injuries in 86 patients with Segond fractures

Structure	Rupture/tear	Edema/contusion	Normal
ACL	76, 88.4%	10, 11.6%	0, 0%
ALL^#^	20, 23.3%	66, 76.7%	0, 0%
ITB*	38, 44.2%†	-	48, 55.8%
PCL	3, 3.5%	7, 8.1%	76, 88.4%
MCL	16, 18.6%	25, 29.1%	45, 52.3%
LCL	3, 3.5%	18, 20.9%	65, 75.6%
MM	18, 20.9%	7, 8.1%	61, 70.9%
LM	31, 36.0%	8, 9.3%	47, 54.7%

^*^Defined as involvement of the capsulo-osseous layer adjacent to the ITB, with the ITB proper intact

^†^The 38 patients represent those with combined ALL + capsulo-osseous ITB involvement

^#^ALL injury categories correspond to Muramatsu classification [17]: tear = Type C (discontinuity), edema/contusion = Type B (warping/thinning/iso-signal), normal = Type A (continuous, clearly defined low-signal band)

Apparent ITB-related involvement was defined as extension of the fracture fragment toward the Gerdy tubercle region with signal abnormality in the capsulo-osseous layer adjacent to the ITB, while the main ITB proper remained structurally continuous without evidence of complete avulsion or discontinuity. In equivocal cases, final classification was determined by consensus of at least two of three readers. This distinction was intentionally made to differentiate true ITB avulsion (rare) from fracture extension into the ITB-adjacent capsular tissues (common).

### Statistical analysis

All statistical analyses were performed using SPSS software (version 27.0; IBM Corp., Armonk, NY, USA). Continuous variables are presented as mean ± standard deviation (SD), and categorical variables as counts and percentages. The Shapiro–Wilk test was used to assess normality of continuous variables. Since most continuous variables were non-normally distributed, the Mann–Whitney U test was used for two-group comparisons. Post-hoc power analysis indicated adequate power (0.82) for the observed difference (effect size 0.80, α = 0.05). Intraobserver and interobserver reliability were assessed using intraclass correlation coefficients (ICC) for continuous variables and Cohen's kappa for categorical variables, with values interpreted as: < 0.40 poor, 0.40–0.59 fair, 0.60–0.74 good, 0.75–1.00 excellent. Receiver operating characteristic (ROC) curve analysis was performed to evaluate the diagnostic performance of fragment size parameters and distance to the Gerdy tubercle for predicting combined ALL + capsulo-osseous ITB involvement. The area under the curve (AUC) was calculated with 95% confidence intervals (CI). The optimal cutoff value was determined using the Youden index (maximum sensitivity + specificity – 1). Sensitivity, specificity, positive predictive value (PPV), and negative predictive value (NPV) were calculated for the identified cutoff. All statistical tests were two-sided, and a p-value < 0.05 was considered statistically significant.

### Language editing

During the preparation of this manuscript, the authors used DeepSeek (a large language model, https://deepseek.com) solely for grammar and syntax refinement. No AI tool was used for data analysis, interpretation, or generation of scientific content. The authors take full responsibility for the accuracy, originality, and integrity of the work.

## Results

### Reliability assessment

Interobserver reliability for CT measurements was excellent for fragment dimensions (ICC range 0.81–0.93) and distance to Gerdy tubercle (ICC = 0.88). Intraobserver reliability was also excellent (ICC range 0.85–0.94). Interobserver agreement for MRI-based classification of ALL and ITB involvement was good to excellent (κ = 0.79 for ALL attachment; κ = 0.82 for combined ITB-related involvement).

### Associated soft-tissue injuries

MRI demonstrated ACL injury in all 86 patients (100%), including 76 complete ruptures (88.4%) and 10 partial tears/contusions (11.6%). Imaging evidence of ALL attachment to the Segond fragment was identified in 86 patients (100%). In 38 patients (44.2%), the fracture fragment extended toward the Gerdy tubercle region, with MRI findings indicating involvement of the capsulo-osseous layer adjacent to the ITB. In all such cases, the ITB proper remained structurally continuous without evidence of true avulsion.

Other associated injuries included MCL injuries (41/86, 47.7%), lateral meniscus tears (31/86, 36.0%), medial meniscus tears (18/86, 20.9%), LCL injuries (21/86, 24.4%), and PCL injuries (10/86, 11.6%). The detailed distribution is presented in Table 1. These associated injuries showed no statistically significant correlation with fragment morphology or location and were therefore not the focus of subsequent analyses.

### Comparison of fragment characteristics between groups

Based on MRI-defined attachment patterns, patients were categorized into two groups:

- *Group 1 (ALL-only, n* = *48)*: fracture fragment associated with the ALL without extension toward the Gerdy tubercle.
- *Group 2 (combined, n* = *38)*: fragment associated with both the ALL and the capsulo-osseous layer adjacent to the ITB (extension toward Gerdy tubercle)

Fragment size parameters, including height, length, width, and estimated volume, did not differ significantly between the two groups (all p > 0.05; Table 2). In contrast, the distance from the anterior margin of the Segond fragment to the center of the Gerdy tubercle was significantly shorter in the combined group (9.2 ± 1.6 mm) compared to the ALL-only group (11.2 ± 3.1 mm; *p* = 0.029; Table 2).

**Table 2:** Comparison of fragment characteristics between ALL-only and combined involvement groups

Parameter	ALL-only (*n* = 48)	ALL + ITB (*n* = 38)	Z	*P-value*
Height (mm)	8.5 ± 1.7	8.7 ± 1.5	−0.434	0.664
Length (mm)	12.1 ± 3.3	12.3 ± 3.3	−0.293	0.769
Width (mm)	1.7 ± 0.6	1.7 ± 0.7	−0.035	0.972
Volume (mm^3^)	177.9 ± 97.3	186.9 ± 107.8	−0.188	0.851
Distance from Gerdy tubercle (mm)	11.2 ± 3.1	9.2 ± 1.6	−2.182	0.029

### Diagnostic performance of fragment distance

**Fig. 2:**
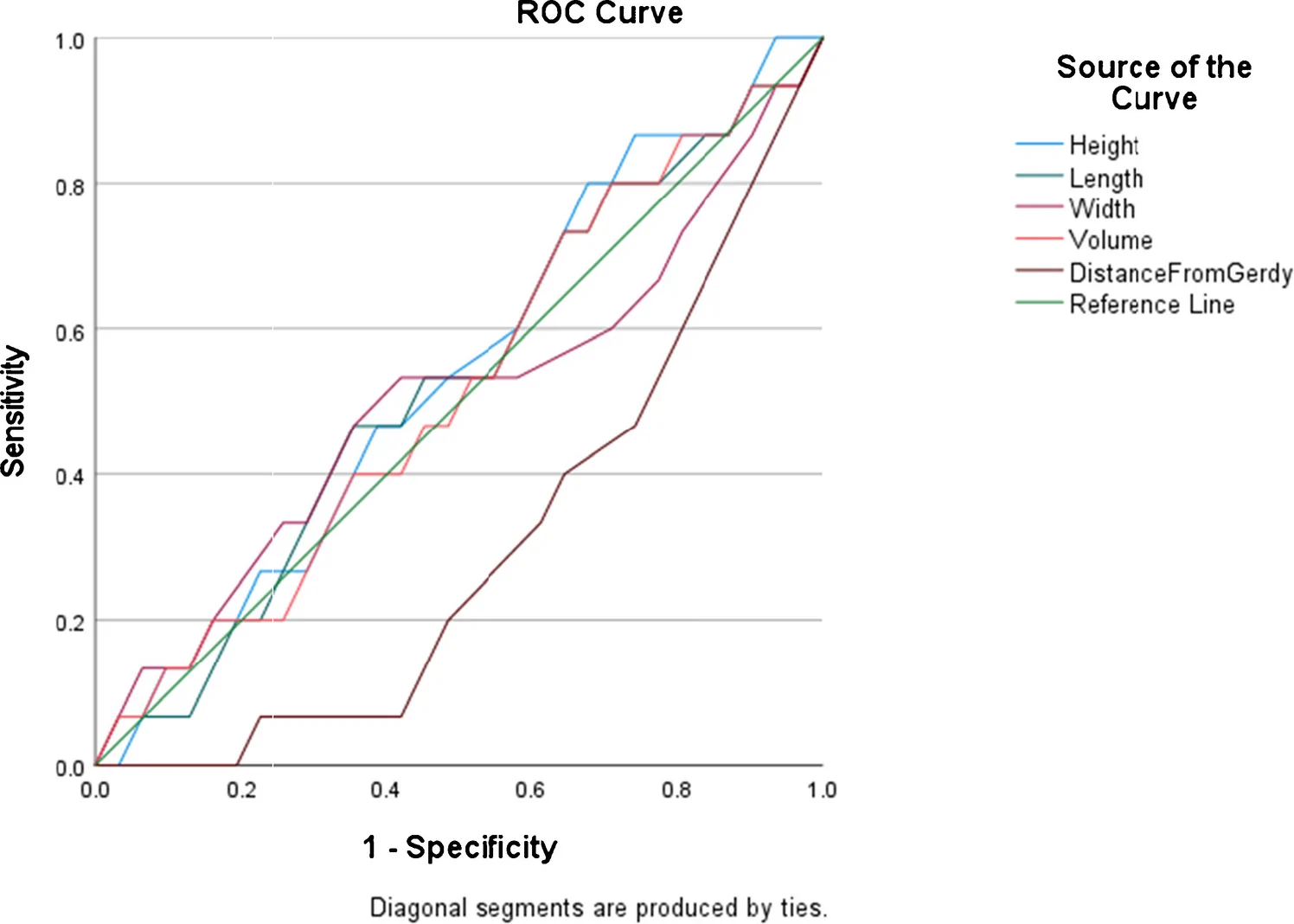
Receiver operating characteristic (ROC) curve analysis of the distance from Segond fracture fragment to the Gerdy tubercle for predicting combined ALL + capsulo-osseous ITB involvement. The area under the curve (AUC) is 0.72 (95% CI: 0.60–0.84). The optimal cutoff distance of < 10 mm (Youden index) yields 78.9% sensitivity and 62.5% specificity

ROC curve analysis demonstrated that the distance from the fragment to the Gerdy tubercle had moderate discriminative ability for identifying combined capsulo-osseous ITB involvement (Fig. 2). The area under the curve (AUC) was 0.72 (95% CI: 0.60–0.84).

Using the Youden index, the optimal cutoff value was determined as < 10 mm. At this threshold:

- Sensitivity: 78.9% (95% CI: 62.7–89.4%)
- Specificity: 62.5% (95% CI: 47.9–75.1%)
- Positive predictive value: 62.5%
- Negative predictive value: 78.9%

Fragment size parameters (height, length, width, volume) showed no significant discriminative ability for predicting combined involvement as confirmed by MRI (AUC range 0.45–0.52, all *p* > 0.05).

### Representative cases

**Fig. 3:**
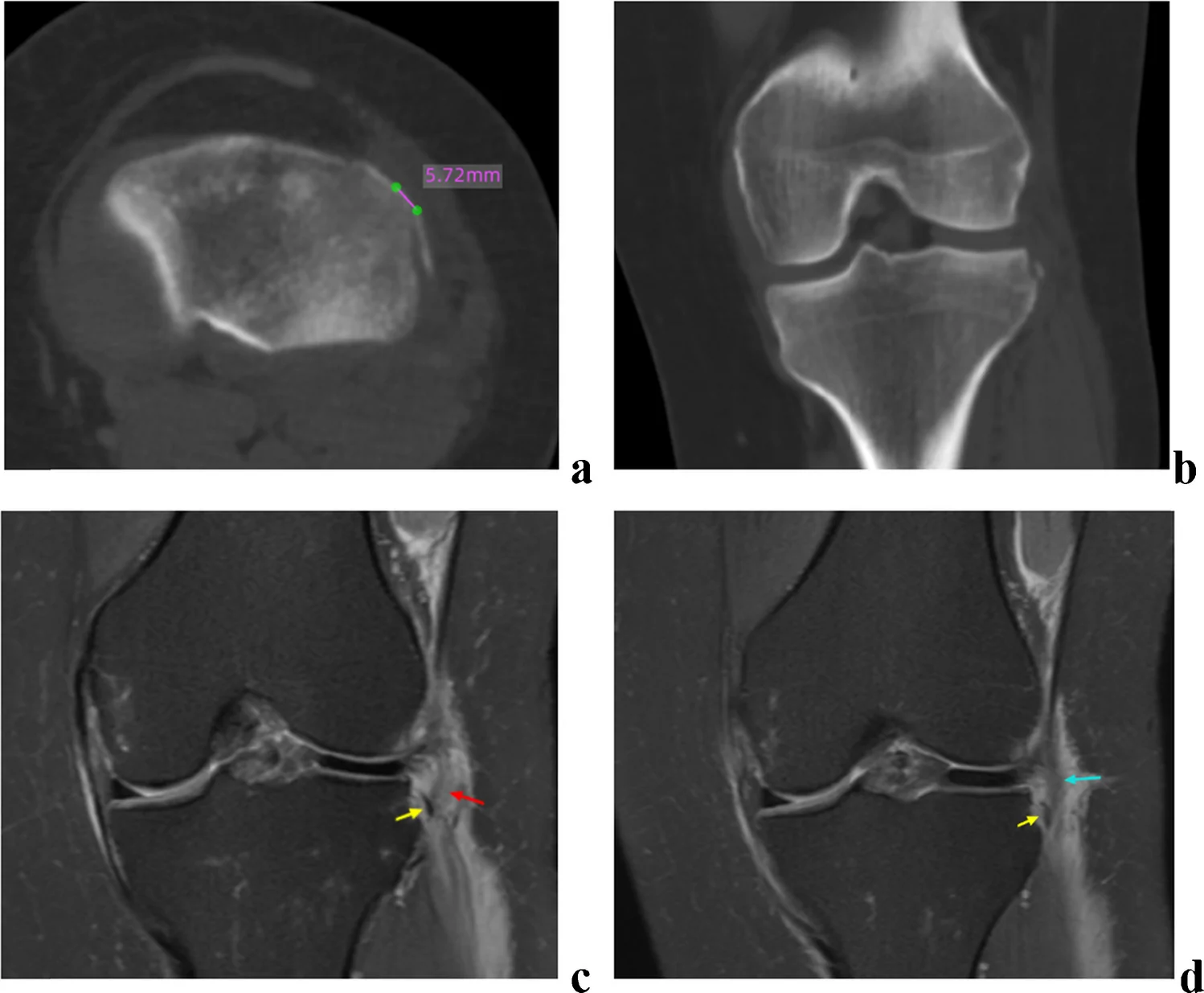
A 29-year-old female patient with a Segond fracture of the left knee. **a** Coronal and (**b**) axial CT images demonstrate an avulsion fracture at the lateral margin of the tibial plateau.The distance from the anterior edge of the fracture fragment to the center of the Gerdy tubercle is 5.72 mm. **c** Coronal fat-suppressed proton density-weighted MR image shows the anterolateral ligament (ALL, red arrow) attached to the avulsed fragment (yellow arrow). **d** Coronal fat-suppressed proton density-weighted MR image at a slightly more anterior plane demonstrates the iliotibial band (ITB, blue arrow) overlying the fragment, with the ITB proper remaining continuous

Figure 3 illustrates a representative case of Segond fracture with combined ALL and capsulo-osseous ITB involvement, demonstrating close fragment proximity to the Gerdy tubercle (5.72 mm).

**Fig. 4:**
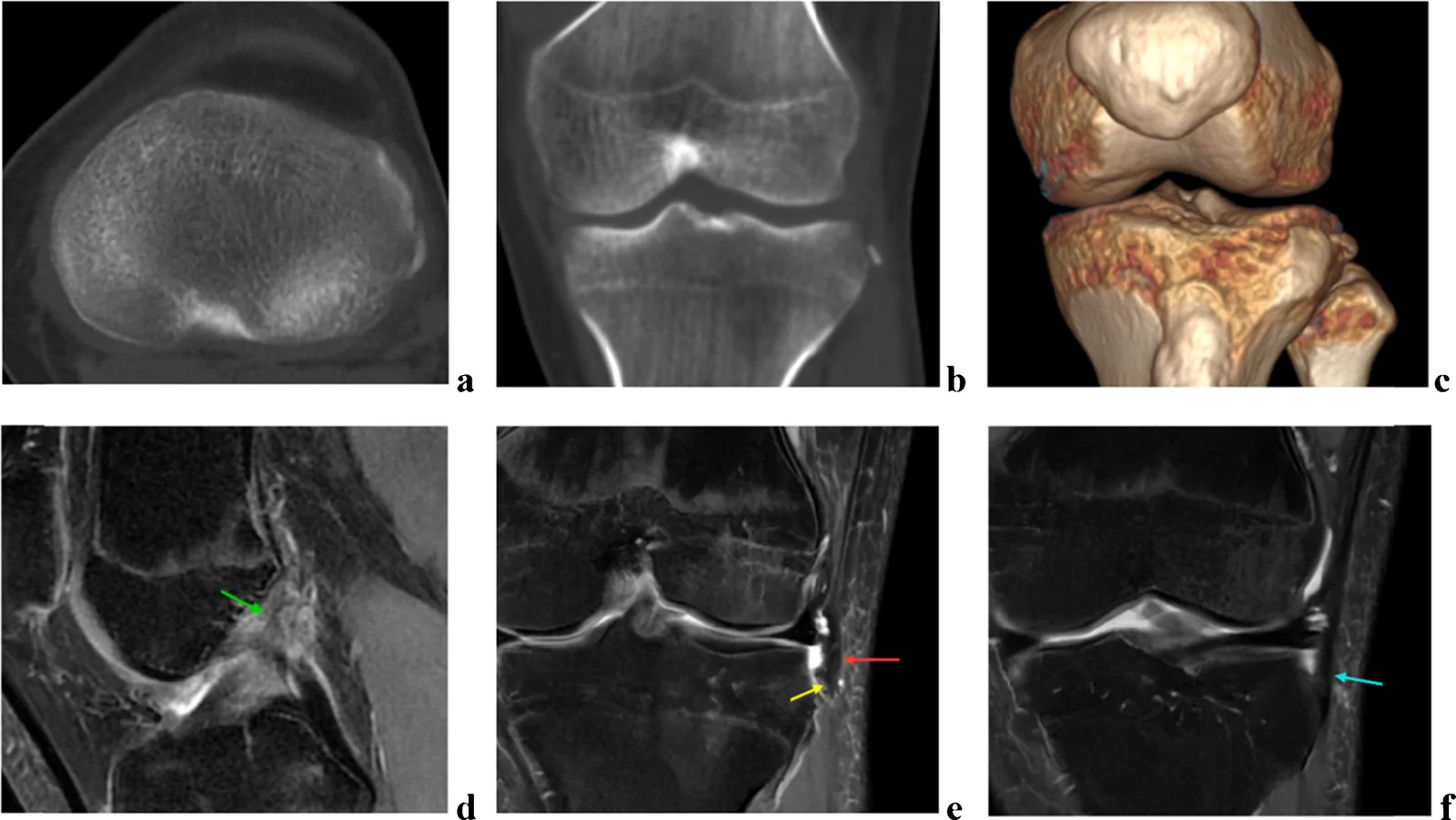
A 17-year-old male patient with a Segond fracture of the left knee demonstrating isolated ALL involvement (ALL-only). **a** Axial, **b** Coronal, and (**c**) Three-dimensional volume-rendered CT images show an avulsion fracture at the lateral margin of the tibial plateau. **d** Sagittal T2-weighted MR image reveals a contusion of the anterior cruciate ligament (ACL, green arrow). **e** Coronal fat-suppressed proton density-weighted MR image demonstrates the anterolateral ligament (ALL, red arrow) attached to the avulsed fragment (yellow arrow). (f) Coronal fat-suppressed proton density-weighted MR image at a more anterior plane shows the iliotibial band (ITB, blue arrow) to be intact, with no associated signal abnormality in the adjacent capsulo-osseous layer

Figure 4 shows an ALL-only case with the fragment located further from the Gerdy tubercle.

## Discussion

The most important finding of this study is that the distance from a Segond fracture fragment to the Gerdy tubercle, measured on preoperative CT, predicts combined involvement of both the ALL and the capsulo-osseous layer adjacent to the ITB. A cutoff of < 10 mm provides moderate diagnostic accuracy (AUC 0.72) with 78.9% sensitivity and 62.5% specificity. Fragment size, in contrast, does not predict injury pattern. These findings may have potential clinical relevance for preoperative assessment of surgical planning in patients with ACL tears and associated Segond fractures, as the CT measurement may provide an early indicator of combined injury patterns that can be confirmed by MRI..

In this series, 100% of Segond fractures demonstrated MRI evidence of ALL attachment to the avulsed fragment. This finding strongly supports the current understanding that Segond fractures represent a bony avulsion of the distal attachment of the ALL. The nearly universal association with ACL injury (100%) further underscores the clinical relevance, as both injuries share a common mechanism of combined varus and internal rotation stress [2]. Our findings align with previous anatomical and biomechanical studies. Claes et al. [5] first demonstrated that the ALL inserts approximately 4–5 mm posterior to the Gerdy tubercle, precisely where Segond fractures occur. Kennedy et al. [12] showed that the ALL functions as a secondary restraint to internal tibial rotation, particularly in ACL-deficient knees, explaining the high rate of combined injuries.

A novel contribution of this study is the quantitative characterization of the subset of Segond fractures that extend toward the Gerdy tubercle and involve the capsulo-osseous layer adjacent to the ITB(44.2%). Helito et al. [10] have also reported a high prevalence of ALL involvement in Segond fractures, with the ITB affected in a subset. This rate should be considered in the context of broader injury patterns. Lintin et al. [18] evaluated 200 consecutive MRI scans in acute knee trauma and found ALL injury in 37.5% of cases, with associated injuries including ACL rupture, ITB sprain, and MCL injury. In the adolescent population, Helito et al. [19] reported ALL injuries in 60.2% of acute ACL injuries, with Segond fractures comprising only 2.9% of cases. These findings highlight that combined anterolateral injuries are common across all age groups and underscore the clinical importance of accurate preoperative assessment of the ALC. This finding challenges the notion that Segond fractures can represent primary ITB avulsion and instead supports the interpretation that apparent ITB involvement reflects spatial fracture extension into the region where the ITB's capsulo-osseous layer attaches to the lateral tibial plateau. This distinction has clinical relevance. In a cadaveric study, Terry et al. [20] described the capsulo-osseous layer of the ITB as a distinct structure that reinforces the lateral capsule. The Gerdy tubercle is the primary insertion of the ITB proper, but the capsulo-osseous layer extends posteriorly and blends with the lateral capsule and the ALL region. Therefore, when a Segond fracture fragment extends anteriorly close to the Gerdy tubercle, it may involve this capsulo-osseous layer—explaining why MRI shows signal abnormality adjacent to the ITB—without implying disruption of the ITB itself.

The distance cutoff of < 10 mm from the fragment to the Gerdy tubercle provides a reproducible, quantitative imaging marker that can be obtained on routine preoperative CT. However, the moderate diagnostic performance (AUC 0.72, specificity 62.5%) warrants cautious interpretation. This CT marker should be used as a screening adjunct rather than a definitive diagnostic test. Surgeons should be aware that fragments < 10 mm may indicate combined ITB involvement, while those ≥ 10 mm suggest isolated ALL avulsion. Currently, there is no consensus on whether ACL reconstruction alone suffices or whether additional anterolateral procedures (e.g., ALL reconstruction, lateral extra-articular tenodesis, or Segond fragment fixation) are required in patients with ACL tears and Segond fractures [15, 21]. A recent international consensus on indications for lateral extra-articular procedures (LEAPs) highlighted this ongoing uncertainty: while strong consensus supported LEAPs in specific high-risk scenarios (young active patients with hamstring autografts, grade 3 pivot shift), statements regarding imaging signs of anterolateral injury—including Segond fractures—did not reach consensus [22]. This underscores the need for more objective, quantitative imaging markers to guide patient selection. Ferretti et al. [15] reported favorable outcomes following direct repair of Segond fractures combined with ACL reconstruction in selected patients with high-grade pivot shift. Our findings suggest that fragment location may help stratify patients: those with fragments close to the Gerdy tubercle have more extensive ALC involvement and might benefit from consideration of additional anterolateral stabilization, whereas those with more posterior fragments (further from Gerdy) may have isolated ALL injury.

The 100% incidence of ACL injury in our series is at the upper end of previously reported ranges (71–100%) [23, 24], likely reflecting our strict inclusion criteria. Similarly, the 100% ALL attachment rate is consistent with Claes et al. [5], who demonstrated ALL attachment in 100% of Segond fractures in a cadaveric series. Regarding ITB involvement, previous studies have reported variable rates. Some authors have suggested that the ITB is the primary structure avulsed [14], while others have found that the ITB remains intact and the fracture lies deep to it [5, 12]. Our findings reconcile these observations by showing that while the ITB proper remains intact, its adjacent capsulo-osseous layer is involved in a substantial subset (44.2%)—and this involvement is predicted by fragment proximity to the Gerdy tubercle.

Several limitations should be acknowledged. First, this was a single-center retrospective study, which may limit generalizability. Second, the absence of surgical or anatomical gold standard validation is a major limitation. MRI, while the best available non-invasive reference standard for soft-tissue evaluation, cannot be considered a definitive gold standard for ligamentous attachment patterns. Our classification of ALL and ITB involvement was based on consensus MRI interpretation by experienced radiologists, not on direct surgical exploration. Consequently, the proposed CT threshold of < 10 mm is validated only against MRI interpretation, not against surgical findings. While we intentionally defined "apparent ITB-related involvement" conservatively to avoid overinterpretation, some misclassification may have occurred. Future prospective studies with surgical correlation are needed to validate our imaging criteria. Third, fragment volume was estimated using a simplified geometric approximation (height × length × width), which does not account for irregular fragment morphology. Fourth, the modest AUC (0.72) indicates that distance alone is not a perfect predictor, and other factors (e.g., fragment orientation, soft-tissue interposition) may also influence injury patterns. Fifth, the limited sample size and event numbers precluded multivariable analysis. Larger future studies are needed to identify independent predictors of combined injury patterns. Finally, we did not assess clinical outcomes, so the impact of fragment location on postoperative results remains unknown.

In conclusion, a Segond fracture fragment distance of < 10 mm from the Gerdy tubercle on preoperative CT is associated with combined involvement of the anterolateral ligament and the capsule-osseous layer of the iliotibial band, as identified on MRI..However, given the moderate diagnostic performance (AUC 0.72) and the lack of surgical validation, this CT marker should be considered an adjunct to—not a replacement for—comprehensive clinical and MRI evaluation. For clinical practice: When this CT finding is present, the imaging report may alert surgeons to possible combined injury, supporting careful evaluation for possible additional anterolateral complex involvement. Conversely, fragments ≥ 10 mm from the Gerdy tubercle suggest isolated ALL avulsion. Prospective studies with surgical correlation are needed to validate these findings before this CT marker can be routinely incorporated into surgical decision-making.

## Data Availability

The data supporting the findings of this study are available from the corresponding author upon reasonable request.

## Abbreviations

MRI: Magnetic resonance imaging
ACL: Anterior cruciate ligament
ALC: Anterolateral complex
ALL: Anterolateral ligament
ITB: Iliotibial bundle
PCL: Posterior cruciate ligament
MCL: Medial collateral ligament
LCL: Lateral collateral ligament
MM: Medial meniscus
LM: Lateral meniscus
SFF: Segond fracture fragment
PACS: picture archiving and communication system
